# Supplementary material for: Analysis of Flavonoid Metabolites in Chaenomeles Petals Using UPLC-ESI-MS/MS
Source: Molecules. 2020 Sep 2;25(17):3994. doi: 10.3390/molecules25173994 (PMC7504807; doi:10.3390/molecules25173994)
Supplement: Supplementary file 1 [file molecules-25-03994-s001.zip › molecules-887120-supplementary-final/molecules-887120-supplementary.pdf]

molecules

MDPI

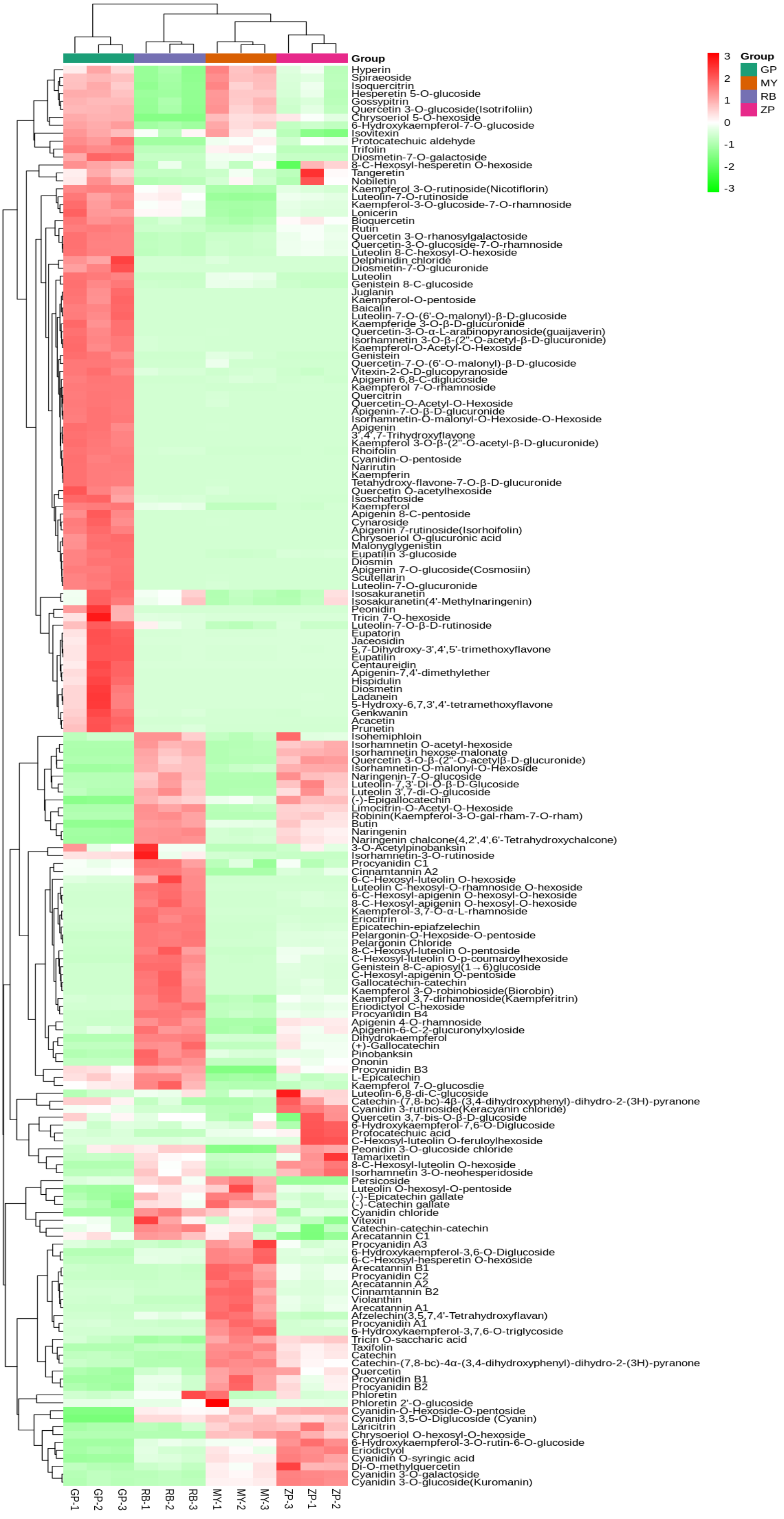

Supplementary Figure 1. Clustering heat map of all flavonoid metabolites. The flavonoid metabolites of four *Chaenomeles* petals was normalized to complete linkage hierarchical clustering. Each example is represented by a column and each metabolite is visualized in a single row. The abundance of each metabolite was visualized in a bar with certain color. With the increase in the abundance value, the color of the bar presented from green to red.

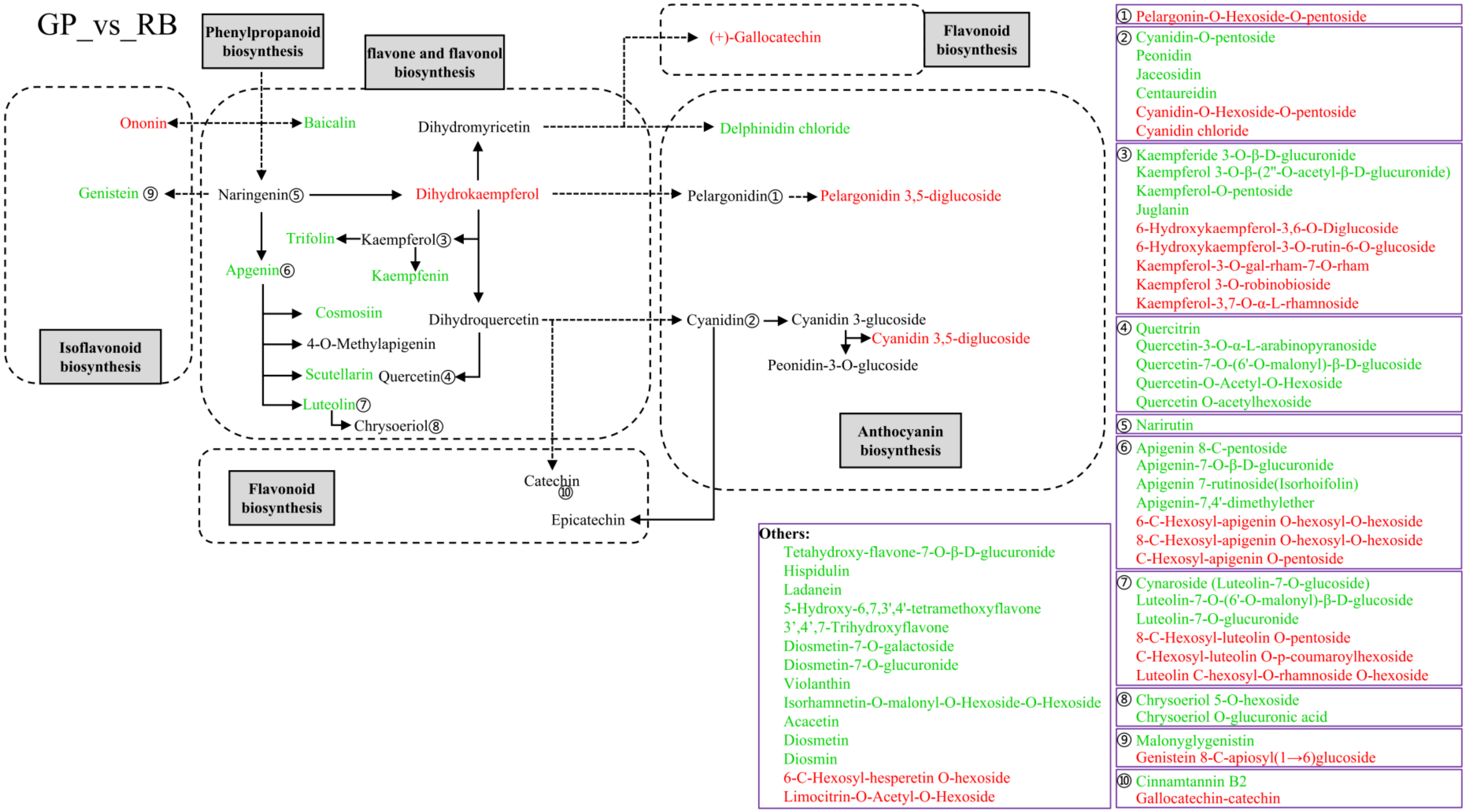
GP_vs_RB
Phenylpropanoid biosynthesis
flavone and flavonol biosynthesis
Flavonoid biosynthesis
Isoflavonoid biosynthesis
Anthocyanin biosynthesis
Flavonoid biosynthesis
(+)-Gallocatechin
Ononin
Baicalin
Dihydromyricetin
Delphinidin chloride
Genistein ⑨
Naringenin⑤
Dihydrokaempferol
Pelargonidin①
Pelargonidin 3,5-diglucoside
Trifolin
Kaempferol③
Apgenin⑥
Kaempfenin
Cosmosiin
Dihydroquercetin
Cyanidin②
Cyanidin 3-glucoside
Cyanidin 3,5-diglucoside
4-O-Methylapigenin
Peonidin-3-O-glucoside
Scutellarin
Quercetin④
Luteolin⑦
Chrysoeriol⑧
Catechin ⑩
Epicatechin
① Pelargonin-O-Hexoside-O-pentoside
② Cyanidin-O-pentoside
Peonidin
Jaceosidin
Centaureidin
Cyanidin-O-Hexoside-O-pentoside
Cyanidin chloride
③ Kaempferide 3-O-β-D-glucuronide
Kaempferol 3-O-β-(2''-O-acetyl-β-D-glucuronide)
Kaempferol-O-pentoside
Juglanin
6-Hydroxykaempferol-3,6-O-Diglucoside
6-Hydroxykaempferol-3-O-rutin-6-O-glucoside
Kaempferol-3-O-gal-rham-7-O-rham
Kaempferol 3-O-robinobioside
Kaempferol-3,7-O-α-L-rhamnoside
④ Quercitrin
Quercetin-3-O-α-L-arabinopyranoside
Quercetin-7-O-(6'-O-malonyl)-β-D-glucoside
Quercetin-O-Acetyl-O-Hexoside
Quercetin O-acetylhexoside
⑤ Narirutin
⑥ Apigenin 8-C-pentoside
Apigenin-7-O-β-D-glucuronide
Apigenin 7-rutinoside(Isorhoifolin)
Apigenin-7,4'-dimethylether
6-C-Hexosyl-apigenin O-hexosyl-O-hexoside
8-C-Hexosyl-apigenin O-hexosyl-O-hexoside
C-Hexosyl-apigenin O-pentoside
⑦ Cynaroside (Luteolin-7-O-glucoside)
Luteolin-7-O-(6'-O-malonyl)-β-D-glucoside
Luteolin-7-O-glucuronide
8-C-Hexosyl-luteolin O-pentoside
C-Hexosyl-luteolin O-p-coumaroylhexoside
Luteolin C-hexosyl-O-rhamnoside O-hexoside
⑧ Chrysoeriol 5-O-hexoside
Chrysoeriol O-glucuronic acid
⑨ Malonylglygenistin
Genistein 8-C-apiosyl(1→6)glucoside
⑩ Cinnamtannin B2
Gallocatechin-catechin
Others:
Tetahydroxy-flavone-7-O-β-D-glucuronide
Hispidulin
Ladanein
5-Hydroxy-6,7,3',4'-tetramethoxyflavone
3',4',7-Trihydroxyflavone
Diosmetin-7-O-galactoside
Diosmetin-7-O-glucuronide
Violanthin
Isorhamnetin-O-malonyl-O-Hexoside-O-Hexoside
Acacetin
Diosmetin
Diosmin
6-C-Hexosyl-hesperetin O-hexoside
Limocitrin-O-Acetyl-O-Hexoside

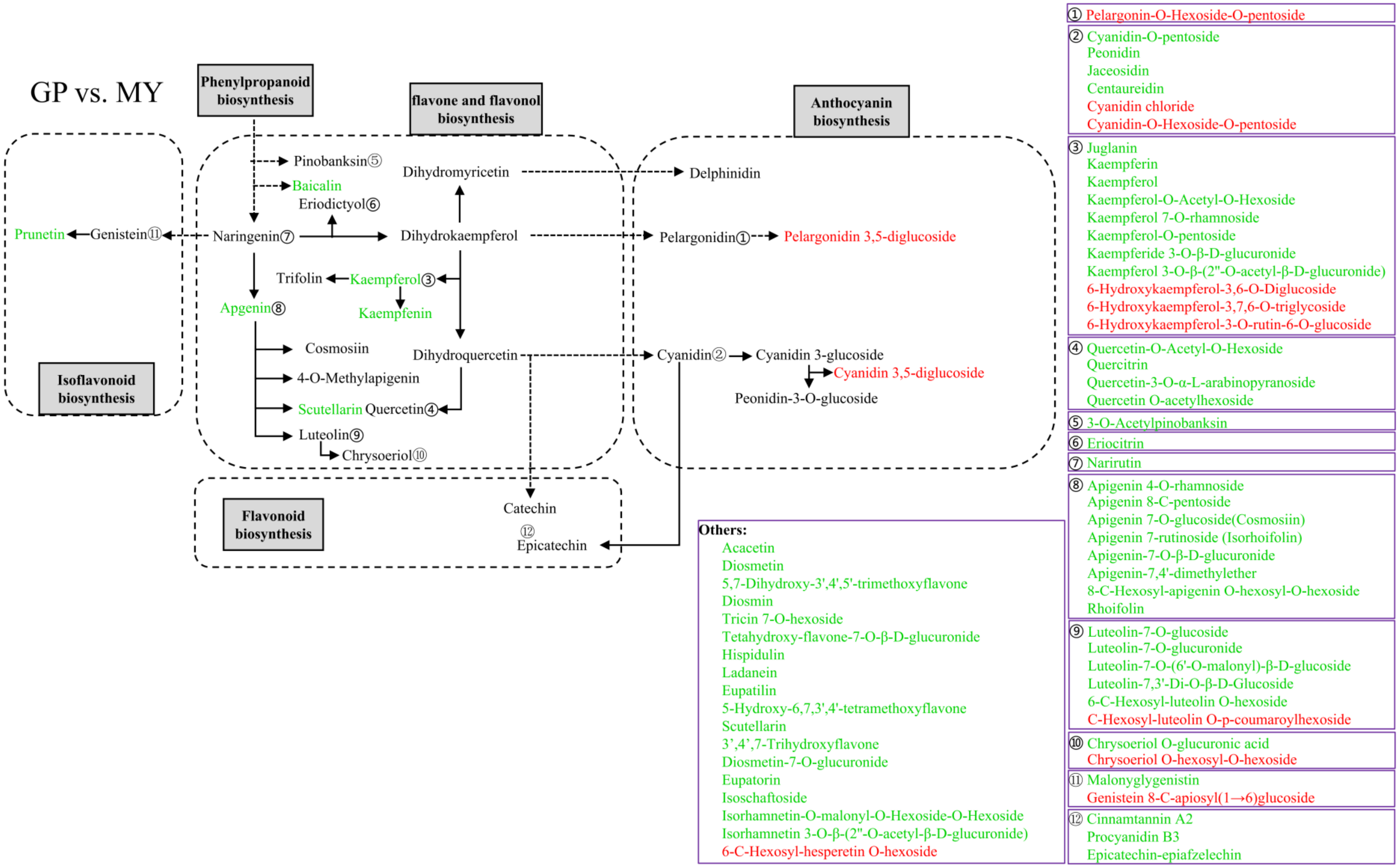
GP vs. MY
Phenylpropanoid biosynthesis
flavone and flavonol biosynthesis
Anthocyanin biosynthesis
Isoflavonoid biosynthesis
Flavonoid biosynthesis
Pinobanksin⑤
Baicalin
Eriodictyol⑥
Dihydromyricetin
Delphinidin
Prunetin
Genistein⑪
Naringenin⑦
Dihydrokaempferol
Pelargonidin①
Pelargonidin 3,5-diglucoside
Trifolin
Kaempferol③
Kaempfenin
Apgenin⑧
Cosmosiin
4-O-Methylapigenin
Scutellarin
Quercetin④
Luteolin⑨
Chrysoeriol⑩
Dihydroquercetin
Cyanidin②
Cyanidin 3-glucoside
Cyanidin 3,5-diglucoside
Peonidin-3-O-glucoside
Catechin
⑫
Epicatechin
Others:
Acacetin
Diosmetin
5,7-Dihydroxy-3',4',5'-trimethoxyflavone
Diosmin
Tricin 7-O-hexoside
Tetahydroxy-flavone-7-O-β-D-glucuronide
Hispidulin
Ladanein
Eupatilin
5-Hydroxy-6,7,3',4'-tetramethoxyflavone
Scutellarin
3',4',7-Trihydroxyflavone
Diosmetin-7-O-glucuronide
Eupatorin
Isoschaftoside
Isorhamnetin-O-malonyl-O-Hexoside-O-Hexoside
Isorhamnetin 3-O-β-(2''-O-acetyl-β-D-glucuronide)
6-C-Hexosyl-hesperetin O-hexoside
① Pelargonin-O-Hexoside-O-pentoside
② Cyanidin-O-pentoside
Peonidin
Jaceosidin
Centaureidin
Cyanidin chloride
Cyanidin-O-Hexoside-O-pentoside
③ Juglanin
Kaempferin
Kaempferol
Kaempferol-O-Acetyl-O-Hexoside
Kaempferol 7-O-rhamnoside
Kaempferol-O-pentoside
Kaempferide 3-O-β-D-glucuronide
Kaempferol 3-O-β-(2''-O-acetyl-β-D-glucuronide)
6-Hydroxykaempferol-3,6-O-Diglucoside
6-Hydroxykaempferol-3,7,6-O-triglycoside
6-Hydroxykaempferol-3-O-rutin-6-O-glucoside
④ Quercetin-O-Acetyl-O-Hexoside
Quercitrin
Quercetin-3-O-α-L-arabinopyranoside
Quercetin O-acetylhexoside
⑤ 3-O-Acetylpinobanksin
⑥ Eriocitrin
⑦ Narirutin
⑧ Apigenin 4-O-rhamnoside
Apigenin 8-C-pentoside
Apigenin 7-O-glucoside(Cosmosiin)
Apigenin 7-rutinoside (Isorhoifolin)
Apigenin-7-O-β-D-glucuronide
Apigenin-7,4'-dimethylether
8-C-Hexosyl-apigenin O-hexosyl-O-hexoside
Rhoifolin
⑨ Luteolin-7-O-glucoside
Luteolin-7-O-glucuronide
Luteolin-7-O-(6'-O-malonyl)-β-D-glucoside
Luteolin-7,3'-Di-O-β-D-Glucoside
6-C-Hexosyl-luteolin O-hexoside
C-Hexosyl-luteolin O-p-coumaroylhexoside
⑩ Chrysoeriol O-glucuronic acid
Chrysoeriol O-hexosyl-O-hexoside
⑪ Malonylgenistin
Genistein 8-C-apiosyl(1→6)glucoside
⑫ Cinnamtannin A2
Procyanidin B3
Epicatechin-epiafzelechin

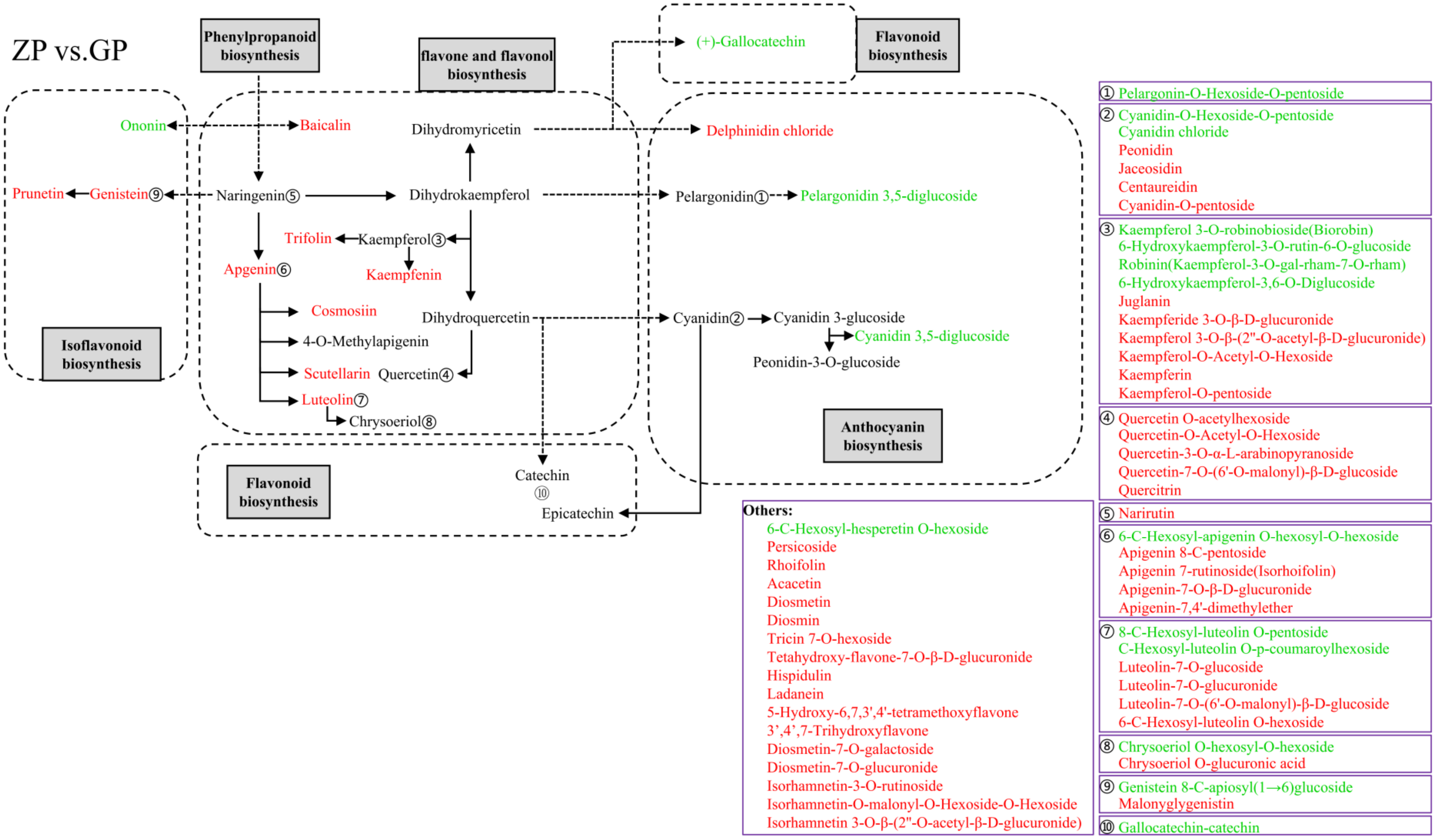
ZP vs.GP
Phenylpropanoid biosynthesis
flavone and flavonol biosynthesis
Flavonoid biosynthesis
(+)-Gallocatechin
Ononin
Baicalin
Dihydromyricetin
Delphinidin chloride
Prunetin
Genistein⑨
Naringenin⑤
Dihydrokaempferol
Pelargonidin①
Pelargonidin 3,5-diglucoside
Trifolin
Kaempferol③
Apgenin⑥
Kaempfenin
Cosmosiin
Dihydroquercetin
Cyanidin②
Cyanidin 3-glucoside
Cyanidin 3,5-diglucoside
4-O-Methylapigenin
Peonidin-3-O-glucoside
Isoflavonoid biosynthesis
Scutellarin
Quercetin④
Luteolin⑦
Chrysoeriol⑧
Anthocyanin biosynthesis
Flavonoid biosynthesis
Catechin
⑩
Epicatechin
Others:
6-C-Hexosyl-hesperetin O-hexoside
Persicoside
Rhoifolin
Acacetin
Diosmetin
Diosmin
Tricin 7-O-hexoside
Tetahydroxy-flavone-7-O-β-D-glucuronide
Hispidulin
Ladanein
5-Hydroxy-6,7,3',4'-tetramethoxyflavone
3',4',7-Trihydroxyflavone
Diosmetin-7-O-galactoside
Diosmetin-7-O-glucuronide
Isorhamnetin-3-O-rutinoside
Isorhamnetin-O-malonyl-O-Hexoside-O-Hexoside
Isorhamnetin 3-O-β-(2''-O-acetyl-β-D-glucuronide)
① Pelargonin-O-Hexoside-O-pentoside
② Cyanidin-O-Hexoside-O-pentoside
Cyanidin chloride
Peonidin
Jaceosidin
Centaureidin
Cyanidin-O-pentoside
③ Kaempferol 3-O-robinobioside(Biorobin)
6-Hydroxykaempferol-3-O-rutin-6-O-glucoside
Robinin(Kaempferol-3-O-gal-rham-7-O-rham)
6-Hydroxykaempferol-3,6-O-Diglucoside
Juglanin
Kaempferide 3-O-β-D-glucuronide
Kaempferol 3-O-β-(2''-O-acetyl-β-D-glucuronide)
Kaempferol-O-Acetyl-O-Hexoside
Kaempferin
Kaempferol-O-pentoside
④ Quercetin O-acetylhexoside
Quercetin-O-Acetyl-O-Hexoside
Quercetin-3-O-α-L-arabinopyranoside
Quercetin-7-O-(6'-O-malonyl)-β-D-glucoside
Quercitrin
⑤ Narirutin
⑥ 6-C-Hexosyl-apigenin O-hexosyl-O-hexoside
Apigenin 8-C-pentoside
Apigenin 7-rutinoside(Isorhoifolin)
Apigenin-7-O-β-D-glucuronide
Apigenin-7,4'-dimethylether
⑦ 8-C-Hexosyl-luteolin O-pentoside
C-Hexosyl-luteolin O-p-coumaroylhexoside
Luteolin-7-O-glucoside
Luteolin-7-O-glucuronide
Luteolin-7-O-(6'-O-malonyl)-β-D-glucoside
6-C-Hexosyl-luteolin O-hexoside
⑧ Chrysoeriol O-hexosyl-O-hexoside
Chrysoeriol O-glucuronic acid
⑨ Genistein 8-C-apiosyl(1→6)glucoside
Malonylglygenistin
⑩ Gallocatechin-catechin

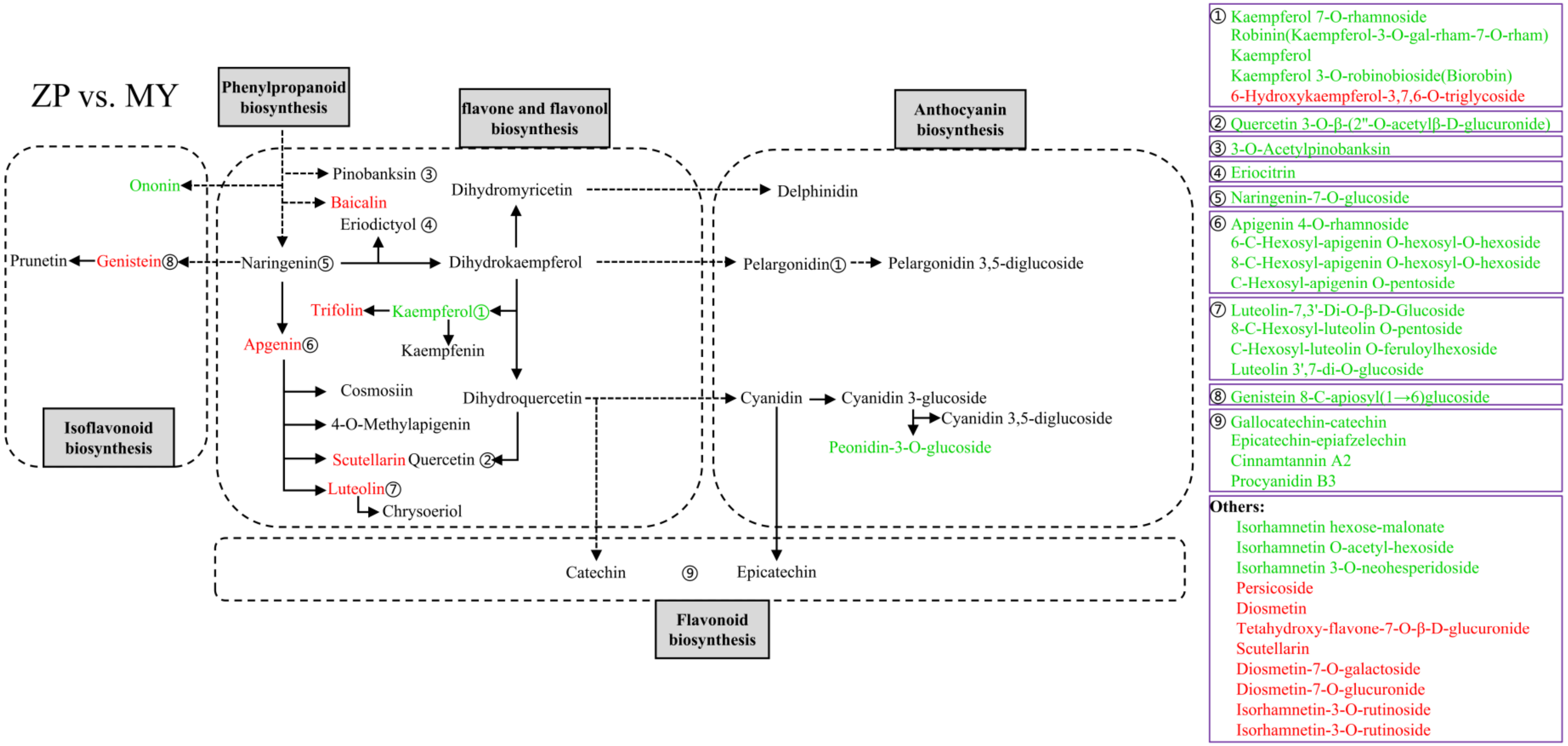
ZP vs. MY
Phenylpropanoid biosynthesis
flavone and flavonol biosynthesis
Anthocyanin biosynthesis
Isoflavonoid biosynthesis
Flavonoid biosynthesis
Pinobanksin ③
Ononin
Baicalin
Eriodictyol ④
Dihydromyricetin
Delphinidin
Prunetin
Genistein⑧
Naringenin⑤
Dihydrokaempferol
Pelargonidin① Pelargonidin 3,5-diglucoside
Trifolin
Kaempferol①
Apgenin⑥
Kaempfenin
Cosmosiin
Dihydroquercetin
Cyanidin
Cyanidin 3-glucoside
Cyanidin 3,5-diglucoside
4-O-Methylapigenin
Peonidin-3-O-glucoside
Scutellarin
Quercetin ②
Luteolin⑦
Chrysoeriol
Catechin
⑨
Epicatechin
① Kaempferol 7-O-rhamnoside
Robinin(Kaempferol-3-O-gal-rham-7-O-rham)
Kaempferol
Kaempferol 3-O-robinobioside(Biorobin)
6-Hydroxykaempferol-3,7,6-O-triglycoside
② Quercetin 3-O-β-(2''-O-acetylβ-D-glucuronide)
③ 3-O-Acetylpinobanksin
④ Eriocitrin
⑤ Naringenin-7-O-glucoside
⑥ Apigenin 4-O-rhamnoside
6-C-Hexosyl-apigenin O-hexosyl-O-hexoside
8-C-Hexosyl-apigenin O-hexosyl-O-hexoside
C-Hexosyl-apigenin O-pentoside
⑦ Luteolin-7,3'-Di-O-β-D-Glucoside
8-C-Hexosyl-luteolin O-pentoside
C-Hexosyl-luteolin O-feruloylhexoside
Luteolin 3',7-di-O-glucoside
⑧ Genistein 8-C-apiosyl(1→6)glucoside
⑨ Gallocatechin-catechin
Epicatechin-epiafzelechin
Cinnamtannin A2
Procyanidin B3
Others:
Isorhamnetin hexose-malonate
Isorhamnetin O-acetyl-hexoside
Isorhamnetin 3-O-neohesperidoside
Persicoside
Diosmetin
Tetahydroxy-flavone-7-O-β-D-glucuronide
Scutellarin
Diosmetin-7-O-galactoside
Diosmetin-7-O-glucuronide
Isorhamnetin-3-O-rutinoside
Isorhamnetin-3-O-rutinoside

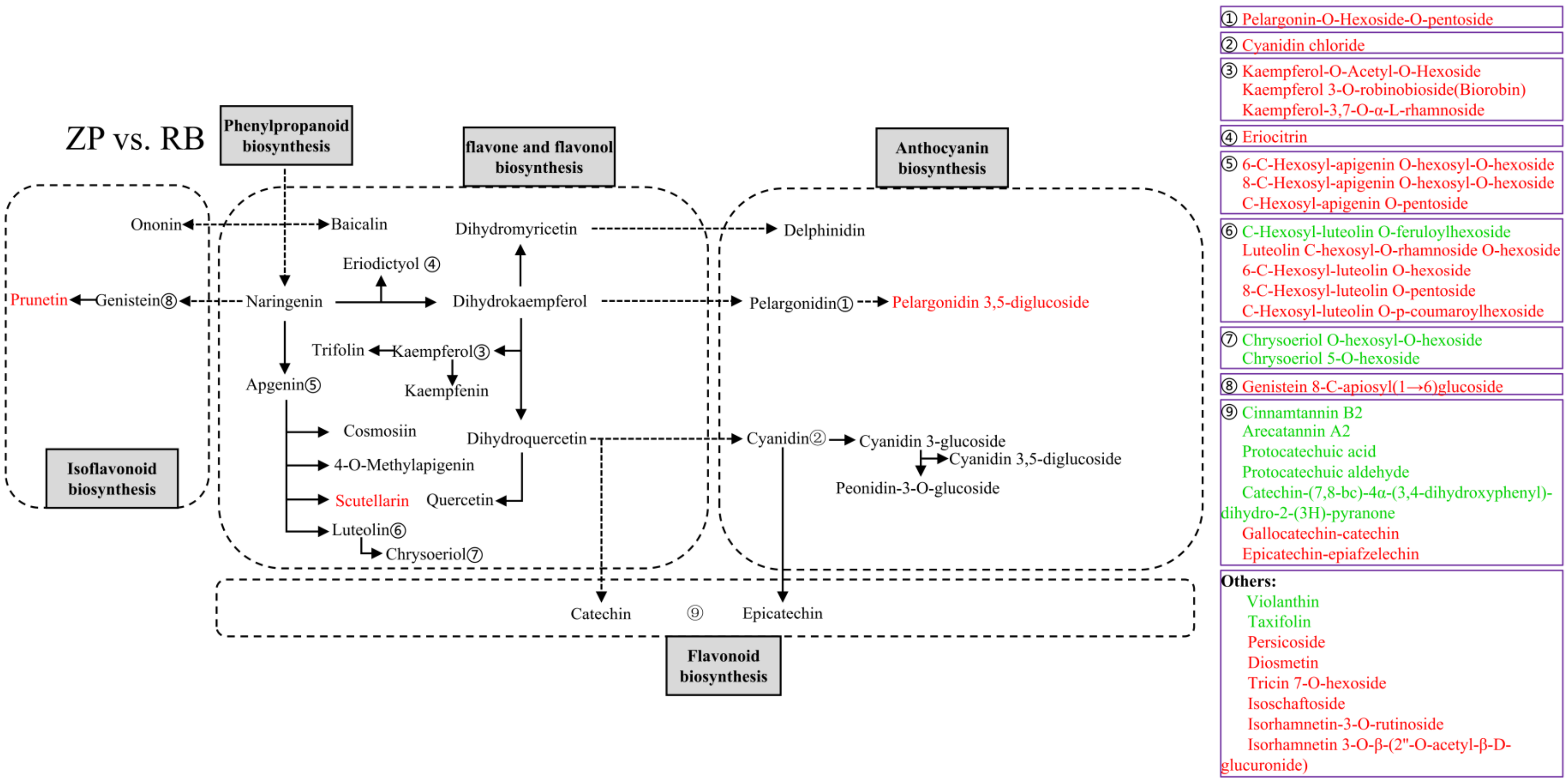

ZP vs. RB
Phenylpropanoid biosynthesis
flavone and flavonol biosynthesis
Anthocyanin biosynthesis
Isoflavonoid biosynthesis
Flavonoid biosynthesis
Ononin
Baicalin
Dihydromyricetin
Delphinidin
Eriodictyol ④
Prunetin
Genistein⑧
Naringenin
Dihydrokaempferol
Pelargonidin①
Pelargonidin 3,5-diglucoside
Trifolin
Kaempferol③
Kaempfenin
Apgenin⑤
Cosmosiin
4-O-Methylapigenin
Scutellarin
Quercetin
Luteolin⑥
Chrysoeriol⑦
Dihydroquercetin
Cyanidin②
Cyanidin 3-glucoside
Cyanidin 3,5-diglucoside
Peonidin-3-O-glucoside
Catechin
⑨
Epicatechin
① Pelargonin-O-Hexoside-O-pentoside
② Cyanidin chloride
③ Kaempferol-O-Acetyl-O-Hexoside
Kaempferol 3-O-robinobioside(Biorobin)
Kaempferol-3,7-O-α-L-rhamnoside
④ Eriocitrin
⑤ 6-C-Hexosyl-apigenin O-hexosyl-O-hexoside
8-C-Hexosyl-apigenin O-hexosyl-O-hexoside
C-Hexosyl-apigenin O-pentoside
⑥ C-Hexosyl-luteolin O-feruloylhexoside
Luteolin C-hexosyl-O-rhamnoside O-hexoside
6-C-Hexosyl-luteolin O-hexoside
8-C-Hexosyl-luteolin O-pentoside
C-Hexosyl-luteolin O-p-coumaroylhexoside
⑦ Chrysoeriol O-hexosyl-O-hexoside
Chrysoeriol 5-O-hexoside
⑧ Genistein 8-C-apiosyl(1→6)glucoside
⑨ Cinnamtannin B2
Arecatannin A2
Protocatechuic acid
Protocatechuic aldehyde
Catechin-(7,8-bc)-4α-(3,4-dihydroxyphenyl)-dihydro-2-(3H)-pyranone
Gallocatechin-catechin
Epicatechin-epiafzelechin
Others:
Violanthin
Taxifolin
Persicoside
Diosmetin
Tricin 7-O-hexoside
Isoschaftoside
Isorhamnetin-3-O-rutinoside
Isorhamnetin 3-O-β-(2''-O-acetyl-β-D-glucuronide)

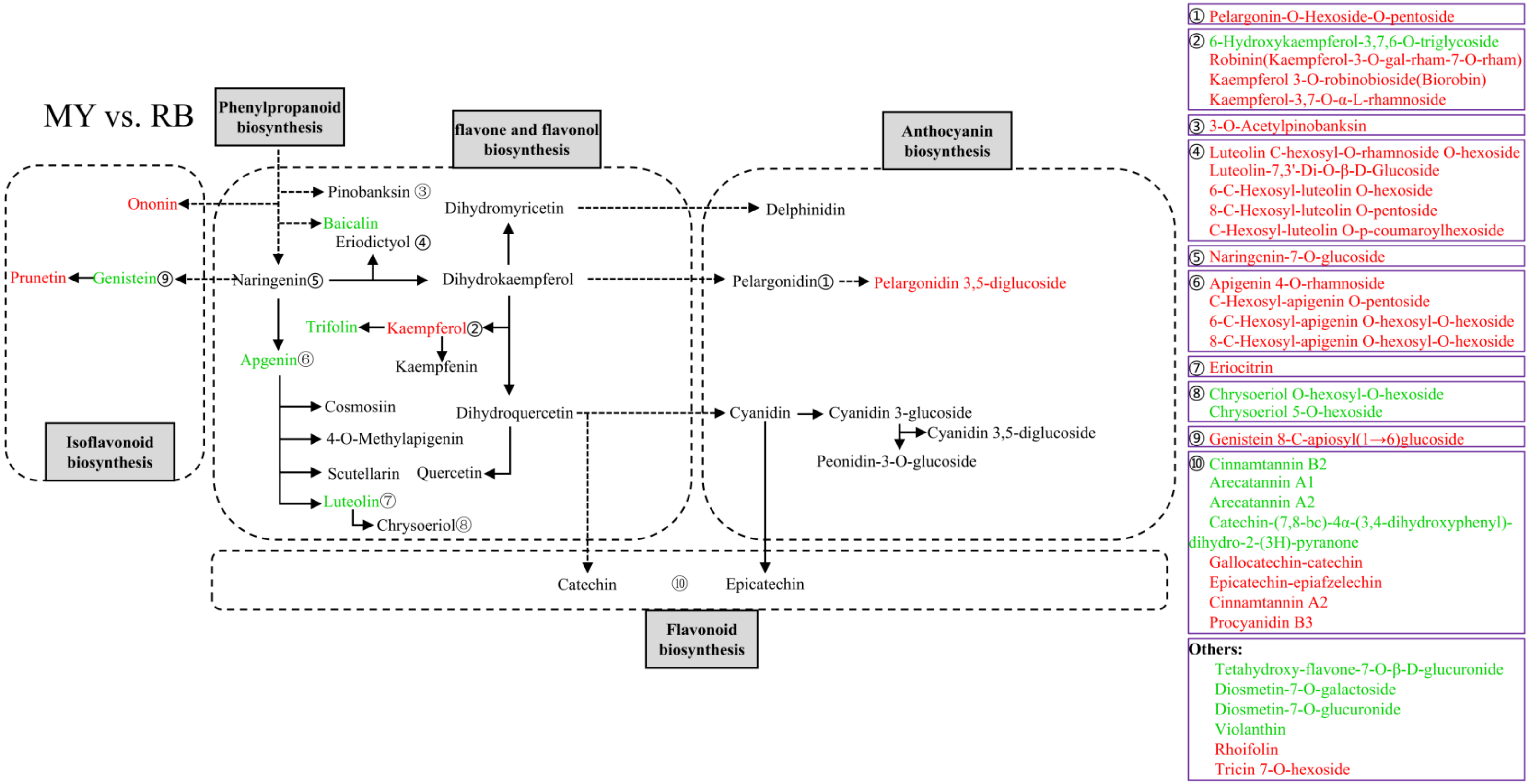

Supplementary Figure 2. The differential flavonoid metabolites in different comparison group, including (a) GP vs. RB, (b) GP vs. MY, (c) ZP vs. GP, (d) ZP vs. MY, (e) ZP vs. RB and (f) MY vs. RB, involved in anthocyanin biosynthesis, flavone and flavonol biosynthesis, and isoflavonoid biosynthesis. Red letters indicate up-regulated when compared with the control group (before vs.), green letters indicate up-regulated when compared with the control group. Unannotated derivatives of flavonoid metabolites by KEGG are listed in the purple box.

a

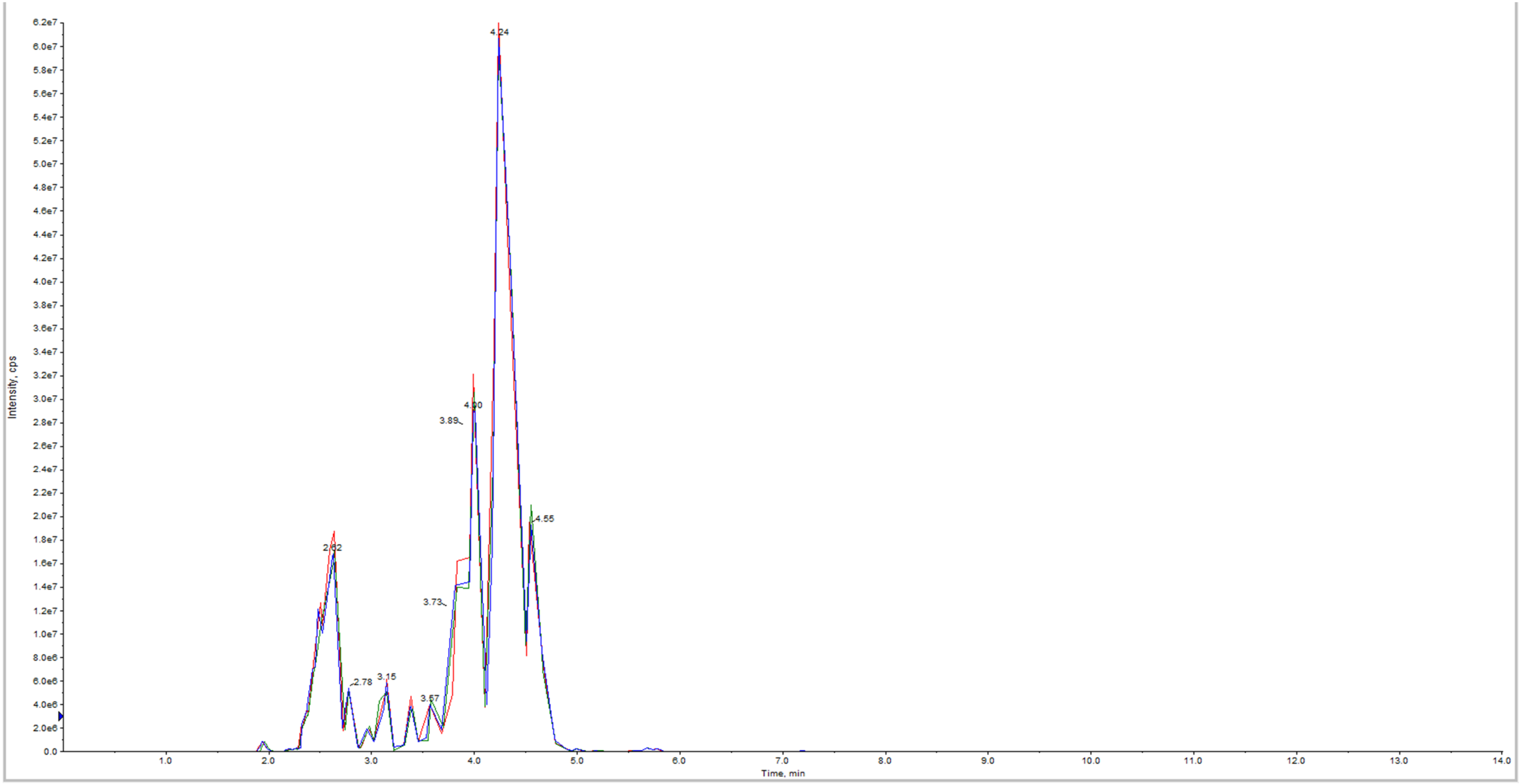

b

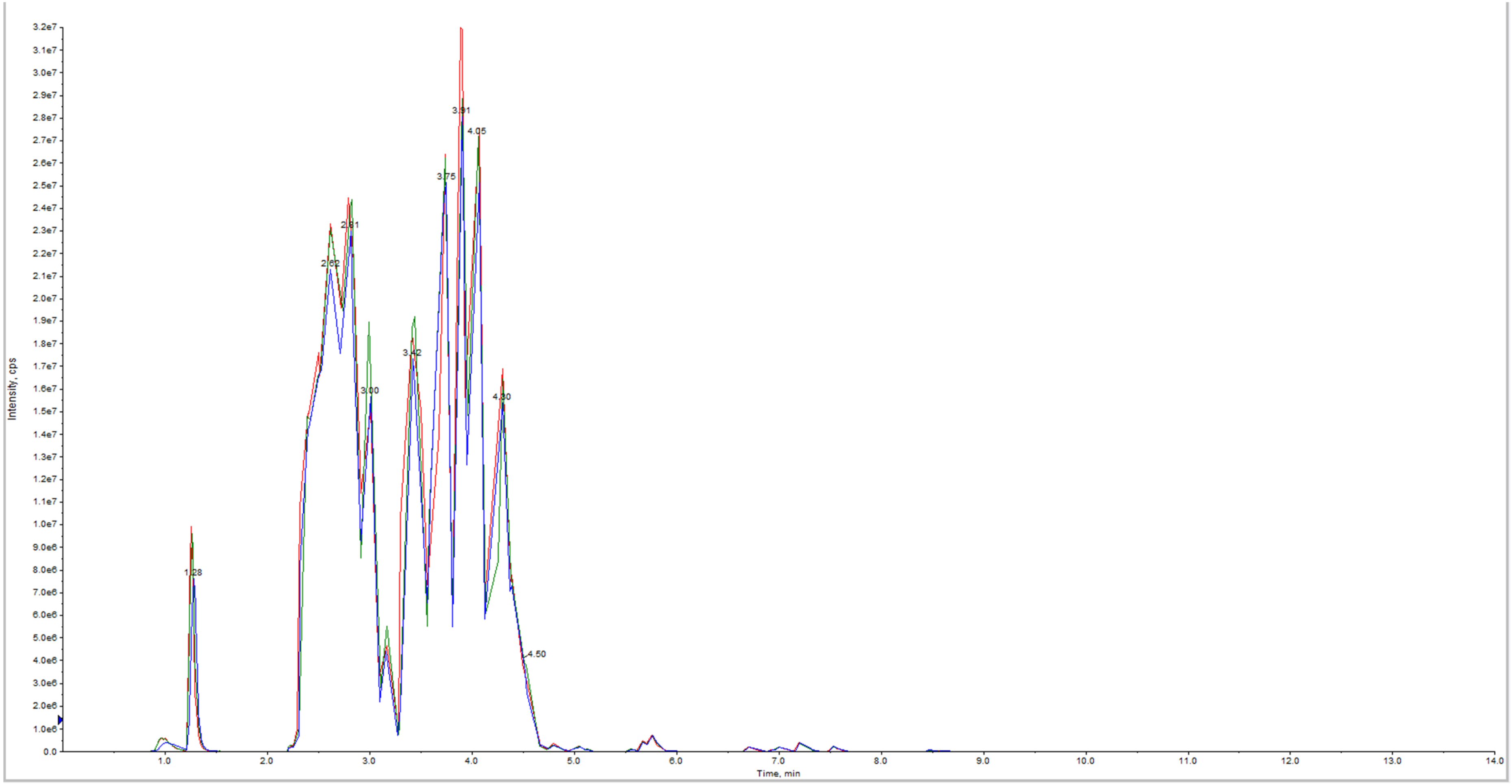

Supplementary Figure S3. The stacking diagram of total ions current (TIC) maps from quality control samples (QC) mass spectrometry. (*a*) TIC of negative ion multiple reaction monitoring (MRM). (*b*) TIC of positive ion MRM.

Supplementary Table 1. A list of the 179 metabolites detected in this study.

Index	Q1 (Da)	Rt (min)	Molecular Weight (Da)	Ionization model	Formula	CAS	Compounds	Class
VHmmp001962	6.11E+02	8.60E+01	6.11E+02	[M]+	C27H31O16+	2611-67-8	Cyanidin 3,5-O-Diglucoside (Cyanin)	Anthocyanins
VHmmp002095	5.95E+02	8.70E+01	6.30E+02	[M-Cl]+	C27H31ClO15	17334-58-6	Pelargonin Chloride	Anthocyanins
VHmmp002395	4.19E+02	8.20E+01	4.18E+02	[M+H]+	C20H18O10	-	Cyanidin-O-pentoside	Anthocyanins
mws0169	5.95E+02	5.60E+01	6.30E+02	[M-Cl]+	C27H31ClO15	18719-76-1	Cyanidin 3-rutinoside(Keracyanin chloride)	Anthocyanins
mws0170	2.87E+02	4.10E+01	2.87E+02	[M]+	C15H11O6	528-58-5	Cyanidin chloride	Anthocyanins
mws1048	4.49E+02	5.70E+01	4.48E+02	[M+H]+	C21H20O11	27661-36-5	Cyanidin 3-O-galactoside	Anthocyanins
mws2210	4.63E+02	5.30E+01	4.98E+02	[M-Cl]+	C22H23ClO11	6906-39-4	Peonidin 3-O-glucoside chloride	Anthocyanins
mws2471	3.03E+02	2.60E+01	3.03E+02	[M]+	C15H11O7	528-53-0	Delphinidin chloride	Anthocyanins
pmb0550	4.49E+02	5.40E+01	4.49E+02	[M]+	C21H21O11	7084-24-4	Cyanidin 3-O-glucoside(Kuromanin)	Anthocyanins
pmb0563	3.01E+02	1.59E+02	3.01E+02	[M]+	C16H13O6	134-01-0	Peonidin	Anthocyanins
pmb2957	4.65E+02	5.50E+01	4.66E+02	[M-H]-	C24H18O10	-	Cyanidin O-syringic acid	Anthocyanins
pmp000004	3.31E+02	7.60E+01	3.30E+02	[M+H]+	C17H14O7	18085-97-7	Jaceosidin	Anthocyanins
pmp000009	3.61E+02	1.63E+02	3.60E+02	[M+H]+	C18H16O8	17313-52-9	Centaureidin	Anthocyanins
mws1140	2.71E+02	1.00E+01	2.72E+02	[M-H]-	C15H12O5	73692-50-9	Naringenin chalcone(4,2',4',6'-Tetrahydroxychalcone)	Chalcones
pme1201	2.73E+02	2.70E+01	2.74E+02	[M-H]-	C15H14O5	60-82-2	Phloretin	Chalcones
mws0064	2.87E+02	1.61E+02	2.88E+02	[M-H]-	C15H12O6	552-58-9	Eriodictyol	Dihydroflavone
mws1034	2.87E+02	1.77E+02	2.86E+02	[M+H]+	C16H14O5	480-43-3	Isosakuranetin(4'-Methylnaringenin)	Dihydroflavone
mws1179	4.33E+02	6.80E+01	4.34E+02	[M-H]-	C21H22O10	529-55-5	Naringenin-7-O-glucoside	Dihydroflavone
mws1454	4.77E+02	1.64E+02	4.78E+02	[M-H]-	C23H26O11	28978-03-2	Persicoside	Dihydroflavone
mws1519	5.95E+02	1.60E+02	5.96E+02	[M-H]-	C27H32O15	13463-28-0	Eriocitrin	Dihydroflavone
pmb3023	4.49E+02	1.62E+02	4.50E+02	[M-H]-	C21H22O11	-	Eriodictyol C-hexoside	Dihydroflavone
pme0376	2.71E+02	1.80E+02	2.72E+02	[M-H]-	C15H12O5	480-41-1	Naringenin	Dihydroflavone
pme3475	2.73E+02	7.40E+01	2.72E+02	[M+H]+	C15H12O5	492-14-8	Butin	Dihydroflavone

pmp001074	2.87E+02	1.76E+02	2.86E+02	[M+H]+	C16H14O5	207-551-8	Isosakuranetin	Dihydroflavone
pmp001078	5.81E+02	1.86E+02	5.80E+02	[M+H]+	C27H32O14	14259-46-2	Narirutin	Dihydroflavone
mws0044	3.03E+02	7.50E+01	3.04E+02	[M-H]-	C15H12O7	480-18-2	Taxifolin	Dihydroflavonol
mws0914	2.71E+02	2.10E+01	2.72E+02	[M-H]-	C15H12O5	548-82-3	Pinobanksin	Dihydroflavonol
mws1094	2.87E+02	1.66E+02	2.88E+02	[M-H]-	C15H12O6	480-20-6	Dihydrokaempferol	Dihydroflavonol
mws1174	3.13E+02	9.30E+01	3.14E+02	[M-H]-	C17H14O6	52117-69-8	3-O-Acetylpinobanksin	Dihydroflavonol
pme1598	4.63E+02	1.12E+02	4.64E+02	[M-H]-	C22H24O11	69651-80-5	Hesperetin 5-O-glucoside	Dihydroflavonol
mws0042	3.05E+02	8.90E+01	3.06E+02	[M-H]-	C15H14O7	970-74-1	(-)-Epigallocatechin	Flavanols
mws0049	3.07E+02	9.10E+01	3.06E+02	[M+H]+	C15H14O7	970-73-0	(+)-Gallocatechin	Flavanols
mws0054	2.89E+02	2.20E+01	2.90E+02	[M-H]-	C15H14O6	154-23-4	Catechin	Flavanols
mws0183	1.53E+02	1.81E+02	1.54E+02	[M-H]-	C7H6O4	99-50-3	Protocatechuic acid	Flavanols
mws0355	4.41E+02	9.00E+01	4.42E+02	[M-H]-	C22H18O10	130405-40-2	(-)-Catechin gallate	Flavanols
mws1397	4.41E+02	8.80E+01	4.42E+02	[M-H]-	C22H18O10	1257-08-5	(-)-Epicatechin gallate	Flavanols
pme0460	2.91E+02	1.90E+01	2.90E+02	[M+H]+	C15H14O6	490-46-0	L-Epicatechin	Flavanols
pme2482	1.39E+02	6.90E+01	1.38E+02	[M+H]+	C7H6O3	139-85-5	Protocatechuic aldehyde	Flavanols
pmn001415	4.51E+02	2.40E+01	4.52E+02	[M-H]-	C24H20O9	-	Catechin-(7,8-bc)-4β-(3,4-dihydroxyphenyl)-dihydro-2-(3H)-pyranone	Flavanols
pmn001416	4.51E+02	2.30E+01	4.52E+02	[M-H]-	C24H20O9	-	Catechin-(7,8-bc)-4α-(3,4-dihydroxyphenyl)-dihydro-2-(3H)-pyranone	Flavanols
mws0043	4.03E+02	1.13E+02	4.02E+02	[M+H]+	C21H22O8	478-01-3	Nobiletin	Flavonoid
mws0047	5.79E+02	6.10E+01	5.78E+02	[M+H]+	C27H30O14	17306-46-6	Rhoifolin	Flavonoid
mws0051	2.85E+02	3.50E+01	2.84E+02	[M+H]+	C16H12O5	480-44-4	Acacetin	Flavonoid
mws0052	4.47E+02	1.23E+02	4.46E+02	[M+H]+	C21H18O11	21967-41-9	Baicalin	Flavonoid
mws0058	2.99E+02	1.68E+02	3.00E+02	[M-H]-	C16H12O6	520-34-3	Diosmetin	Flavonoid
mws0071	4.15E+02	1.43E+02	4.16E+02	[M-H]-	C21H20O9	133538-77-9	Apigenin 4-O-rhamnoside	Flavonoid
mws0129	2.85E+02	1.65E+02	2.84E+02	[M+H]+	C16H12O5	437-64-9	Genkwanin	Flavonoid
mws1073	5.95E+02	1.45E+02	5.94E+02	[M+H]+	C27H30O15	23666-13-9	Apigenin 6,8-C-diglucoside	Flavonoid

mws1474	3.45E+02	1.30E+01	3.44E+02	[M+H]+	C18H16O7	18103-42-9	5,7-Dihydroxy-3',4',5'-trimethoxyflavone	Flavonoid
mws1661	6.09E+02	1.67E+02	6.08E+02	[M+H]+	C28H32O15	520-27-4	Diosmin	Flavonoid
pmb0566	5.81E+02	1.36E+02	5.80E+02	[M+H]+	C26H28O15	-	Luteolin O-hexosyl-O-pentoside	Flavonoid
pmb0588	6.11E+02	4.30E+01	6.10E+02	[M+H]+	C27H30O16	52187-80-1	Luteolin 3',7-di-O-glucoside	Flavonoid
pmb0603	6.25E+02	3.70E+01	6.24E+02	[M+H]+	C28H32O16	-	Chrysoeriol O-hexosyl-O-hexoside	Flavonoid
pmb0665	6.11E+02	1.35E+02	6.10E+02	[M+H]+	C27H30O16	-	Luteolin 8-C-hexosyl-O-hexoside	Flavonoid
pmb0681	4.03E+02	1.44E+02	4.02E+02	[M+H]+	C20H18O9	-	Apigenin 8-C-pentoside	Flavonoid
pmb0691	7.57E+02	1.42E+02	7.56E+02	[M+H]+	C33H40O20	-	Luteolin C-hexosyl-O-rhamnoside O-hexoside	Flavonoid
pmb0736	4.93E+02	1.28E+02	4.92E+02	[M+H]+	C23H24O12	-	Tricin 7-O-hexoside	Flavonoid
pmb2999	4.61E+02	3.60E+01	4.62E+02	[M-H]-	C22H22O11	-	Chrysoeriol 5-O-hexoside	Flavonoid
pmb3006	4.31E+02	4.60E+01	4.32E+02	[M-H]-	C21H20O10	578-74-5	Apigenin 7-O-glucoside(Cosmosiin)	Flavonoid
pmb3007	4.75E+02	3.80E+01	4.76E+02	[M-H]-	C22H20O12	-	Chrysoeriol O-glucuronic acid	Flavonoid
pmb3026	5.05E+02	1.21E+02	5.06E+02	[M-H]-	C23H22O13	-	Quercetin O-acetylhexoside	Flavonoid
pmb3041	5.21E+02	1.29E+02	5.22E+02	[M-H]-	C23H22O14	-	Tricin O-saccharic acid	Flavonoid
pme0368	5.79E+02	6.60E+01	5.78E+02	[M+H]+	C27H30O14	552-57-8	Apigenin 7-rutinoside(Isorhoifolin)	Flavonoid
pmn001639	4.47E+02	1.34E+02	4.48E+02	[M-H]-	C21H20O11	1.27E+06	Cynaroside	Flavonoid
pmn001697	4.45E+02	4.80E+01	4.46E+02	[M-H]-	C21H18O11	-	Apigenin-7-O-β-D-glucuronide	Flavonoid
pmn001702	4.61E+02	1.10E+01	4.62E+02	[M-H]-	C21H18O12	-	Tetahydroxy-flavone-7-O-β-D-glucuronide	Flavonoid
pmn001713	5.93E+02	1.40E+02	5.94E+02	[M-H]-	C27H30O15	-	Luteolin-7-O-β-D-rutinoside	Flavonoid
pmp000001	3.01E+02	1.15E+02	3.00E+02	[M+H]+	C16H12O6	1447-88-7	Hispidulin	Flavonoid
pmp000002	3.15E+02	1.20E+01	3.14E+02	[M+H]+	C17H14O6	10176-71-3	Ladanein	Flavonoid
pmp000006	3.45E+02	6.70E+01	3.44E+02	[M+H]+	C18H16O7	22368-21-4	Eupatilin	Flavonoid
pmp000008	3.59E+02	1.40E+01	3.58E+02	[M+H]+	C19H18O7	21763-80-4	5-Hydroxy-6,7,3',4'-tetramethoxyflavone	Flavonoid
pmp000012	4.63E+02	1.71E+02	4.62E+02	[M+H]+	C21H18O12	27740-01-8	Scutellarin	Flavonoid
pmp000013	5.07E+02	1.79E+02	5.06E+02	[M+H]+	C24H26O12	-	Eupatilin 3-glucoside	Flavonoid
pmp000126	6.11E+02	1.37E+02	6.10E+02	[M+H]+	C27H30O16	29428-58-8	Luteolin-6,8-di-C-glucoside	Flavonoid
pmp000344	2.71E+02	9.20E+01	2.70E+02	[M+H]+	C15H10O5	2150-11-0	3′,4′,7-Trihydroxyflavone	Flavonoid

pmp000571	2.71E+02	4.50E+01	2.70E+02	[M+H]+	C15H10O5	520-36-5	Apigenin	Flavonoid
pmp000572	2.87E+02	4.20E+01	2.86E+02	[M+H]+	C15H10O6	491-70-3	Luteolin	Flavonoid
pmp000578	4.63E+02	4.40E+01	4.62E+02	[M+H]+	C25H18O9	29741-10-4	Luteolin-7-O-glucuronide	Flavonoid
pmp000579	4.63E+02	1.69E+02	4.62E+02	[M+H]+	C22H22O11	-	Diosmetin-7-O-galactoside	Flavonoid
pmp000583	4.77E+02	1.70E+02	4.76E+02	[M+H]+	C22H20O12	35110-20-4	Diosmetin-7-O-glucuronide	Flavonoid
pmp000587	5.35E+02	1.39E+02	5.34E+02	[M+H]+	C24H22O14	-	Luteolin-7-O-(6'-O-malonyl)-β-D-glucoside	Flavonoid
pmp000593	5.95E+02	1.41E+02	5.94E+02	[M+H]+	C27H30O15	3563-98-2	Luteolin-7-O-rutinoside	Flavonoid
pmp000595	6.11E+02	1.38E+02	6.10E+02	[M+H]+	C27H30O16	257-724-7	Luteolin-7,3'-Di-O-β-D-Glucoside	Flavonoid
pmp000786	3.45E+02	1.87E+02	3.44E+02	[M+H]+	C18H16O7	855-96-9	Eupatorin	Flavonoid
pmp001079	5.95E+02	1.48E+02	5.94E+02	[M+H]+	C27H30O15	20633-84-5	Lonicerin	Flavonoid
pmp001204	2.99E+02	4.70E+01	2.98E+02	[M+H]+	C17H14O5	5128-44-9	Apigenin-7,4'-dimethylether	Flavonoid
mws0048	4.31E+02	1.32E+02	4.32E+02	[M-H]-	C21H20O10	3681-93-4	Vitexin	Flavonoid carbonoside
mws1292	5.65E+02	1.75E+02	5.64E+02	[M+H]+	C26H28O14	52012-29-0	Isoschaftoside	Flavonoid carbonoside
mws1434	4.31E+02	1.72E+02	4.32E+02	[M-H]-	C21H20O10	29702-25-8	Isovitexin	Flavonoid carbonoside
pmb0618	6.27E+02	1.01E+02	6.26E+02	[M+H]+	C28H34O16	-	8-C-Hexosyl-hesperetin O-hexoside	Flavonoid carbonoside
pmb0624	6.11E+02	1.50E+01	6.10E+02	[M+H]+	C27H30O16	-	6-C-Hexosyl-luteolin O-hexoside	Flavonoid carbonoside
pmb0626	7.57E+02	9.50E+01	7.56E+02	[M+H]+	C33H40O20	-	6-C-Hexosyl-apigenin O-hexosyl-O-hexoside	Flavonoid carbonoside
pmb0639	7.57E+02	1.00E+02	7.56E+02	[M+H]+	C33H40O20	-	8-C-Hexosyl-apigenin O-hexosyl-O-hexoside	Flavonoid carbonoside
pmb0645	6.27E+02	9.40E+01	6.26E+02	[M+H]+	C28H34O16	-	6-C-Hexosyl-hesperetin O-hexoside	Flavonoid carbonoside
pmb0647	5.81E+02	1.02E+02	5.80E+02	[M+H]+	C26H28O15	-	8-C-Hexosyl-luteolin O-pentoside	Flavonoid carbonoside
pmb0652	5.65E+02	1.05E+02	5.64E+02	[M+H]+	C26H28O14	-	C-Hexosyl-apigenin O-pentoside	Flavonoid carbonoside
pmb0660	7.57E+02	1.04E+02	7.56E+02	[M+H]+	C36H36O18	-	C-Hexosyl-luteolin O-p-coumaroylhexoside	Flavonoid carbonoside
pmb0662	7.87E+02	1.03E+02	7.86E+02	[M+H]+	C37H38O19	-	C-Hexosyl-luteolin O-feruloylhexoside	Flavonoid carbonoside
pmb0663	6.11E+02	9.90E+01	6.10E+02	[M+H]+	C27H30O16	-	8-C-Hexosyl-luteolin O-hexoside	Flavonoid carbonoside
pme1611	4.33E+02	1.78E+02	4.34E+02	[M-H]-	C21H22O10	6.51E+05	Isohemiphloin	Flavonoid carbonoside
pmp000237	5.79E+02	1.46E+02	5.78E+02	[M+H]+	C26H26O15	-	Apigenin-6-C-2-glucuronylxyloside	Flavonoid carbonoside
pmp000411	5.65E+02	1.24E+02	5.64E+02	[M+H]+	C26H28O14	-	Genistein 8-C-apiosyl(1→6)glucoside	Flavonoid carbonoside

pmp000413	4.33E+02	1.25E+02	4.32E+02	[M+H]+	C21H20O10	66026-80-0	Genistein 8-C-glucoside	Flavonoid carbonoside
pmp001106	5.95E+02	1.33E+02	5.94E+02	[M+H]+	C27H30O15	-	Vitexin-2-O-D-glucopyranoside	Flavonoid carbonoside
pmp001111	5.79E+02	1.52E+02	5.78E+02	[M+H]+	C27H30O14	40581-17-7	Violanthin	Flavonoid carbonoside
GQ512002	6.23E+02	1.74E+02	6.24E+02	[M-H]-	C28H32O16	604-80-8	Isorhamnetin-3-O-rutinoside	Flavonols
GQ512005	5.95E+02	5.20E+01	5.94E+02	[M+H]+	C27H30O15	-	Kaempferol-3-O-glucoside-7-O-rhamnoside	Flavonols
GQ512006	6.11E+02	3.10E+01	6.10E+02	[M+H]+	C27H30O16	-	Quercetin-3-O-glucoside-7-O-rhamnoside	Flavonols
Li512112	5.63E+02	6.50E+01	5.64E+02	[M-H]-	C25H24O15	-	Isorhamnetin hexose-malonate	Flavonols
Li512117	6.11E+02	3.20E+01	6.10E+02	[M+H]+	C27H30O16	-	Quercetin 3-O-rhanosylgalactoside	Flavonols
VHmmn003360	5.49E+02	7.70E+01	5.50E+02	[M-H]-	C24H22O15	-	Quercetin-O-Acetyl-O-Hexoside	Flavonols
VHmmn003629	5.33E+02	7.80E+01	5.34E+02	[M-H]-	C24H22O14	-	Kaempferol-O-Acetyl-O-Hexoside	Flavonols
VHmmn003659	5.49E+02	7.90E+01	5.50E+02	[M-H]-	C25H26O14	-	Limocitrin-O-Acetyl-O-Hexoside	Flavonols
VHmmp002008	5.81E+02	8.00E+01	5.80E+02	[M+H]+	C26H28O15	-	Cyanidin-O-Hexoside-O-pentoside	Flavonols
VHmmp002248	5.65E+02	8.10E+01	5.65E+02	[M]+	C26H29O14	-	Pelargonin-O-Hexoside-O-pentoside	Flavonols
VHmmp003135	7.27E+02	8.30E+01	7.26E+02	[M+H]+	C31H34O20	-	Isorhamnetin-O-malonyl-O-Hexoside-O-Hexoside	Flavonols
VHmmp003702	5.65E+02	8.40E+01	5.64E+02	[M+H]+	C25H24O15	-	Isorhamnetin-O-malonyl-O-Hexoside	Flavonols
VHmmp003848	4.19E+02	8.50E+01	4.18E+02	[M+H]+	C20H18O10	-	Kaempferol-O-pentoside	Flavonols
mws0045	4.49E+02	1.18E+02	4.48E+02	[M+H]+	C21H20O11	522-12-3	Quercitrin	Flavonols
mws0055	3.73E+02	1.27E+02	3.72E+02	[M+H]+	C20H20O7	481-53-8	Tangeretin	Flavonols
mws0059	6.09E+02	4.00E+01	6.10E+02	[M-H]-	C27H30O16	153-18-4	Rutin	Flavonols
mws0061	4.63E+02	1.26E+02	4.64E+02	[M-H]-	C21H20O12	482-36-0	Hyperin	Flavonols
mws0089	4.47E+02	1.55E+02	4.48E+02	[M-H]-	C21H20O11	16290-07-6	Kaempferol 7-O-glucosdie	Flavonols
mws0091	4.63E+02	3.00E+01	4.64E+02	[M-H]-	C21H20O12	482-35-9	Isoquercitrin	Flavonols
mws0856	4.63E+02	5.90E+01	4.64E+02	[M-H]-	C21H20O12	20229-56-5	Spiraeoside	Flavonols
mws0913	4.47E+02	1.53E+02	4.48E+02	[M-H]-	C21H20O11	23627-87-4	Trifolin	Flavonols
mws0919	4.31E+02	2.50E+01	4.32E+02	[M-H]-	C21H20O10	482-39-3	Kaempferin	Flavonols
mws1003	3.31E+02	5.80E+01	3.32E+02	[M-H]-	C16H12O8	53472-37-0	Laricitrin	Flavonols
mws1035	7.41E+02	1.14E+02	7.40E+02	[M+H]+	C33H40O19	301-19-9	Robinin(Kaempferol-3-O-gal-rham-7-O-rham)	Flavonols

mws1068	2.85E+02	4.90E+01	2.86E+02	[M-H]-	C15H10O6	520-18-3	Kaempferol	Flavonols
mws1329	4.63E+02	3.40E+01	4.64E+02	[M-H]-	C21H20O12	491-50-9	Gossypitrin	Flavonols
mws2627	3.17E+02	2.00E+01	3.16E+02	[M+H]+	C16H12O7	603-61-2	Tamarixetin	Flavonols
mws4183	4.33E+02	2.80E+01	4.34E+02	[M-H]-	C20H18O11	22255-13-6	Quercetin-3-O-α-L-arabinopyranoside(guaijaverin)	Flavonols
pmb3013	5.19E+02	6.30E+01	5.20E+02	[M-H]-	C24H24O13	-	Isorhamnetin O-acetyl-hexoside	Flavonols
pmb3894	3.29E+02	9.00E+00	3.30E+02	[M-H]-	C17H14O7	6.14E+04	Di-O-methylquercetin	Flavonols
pme0321	4.31E+02	5.10E+01	4.32E+02	[M-H]-	C21H20O10	20196-89-8	Kaempferol 7-O-rhamnoside	Flavonols
pme0369	5.93E+02	6.00E+01	5.94E+02	[M-H]-	C27H30O15	17650-84-9	Kaempferol 3-O-rutinoside(Nicotiflorin)	Flavonols
pme1540	6.25E+02	1.73E+02	6.24E+02	[M+H]+	C28H32O16	55033-90-4	Isorhamnetin 3-O-neohesperidoside	Flavonols
pme1605	5.93E+02	5.00E+01	5.94E+02	[M-H]-	C27H30O15	17297-56-2	Kaempferol 3-O-robinobioside(Biorobin)	Flavonols
pme2493	5.79E+02	1.57E+02	5.78E+02	[M+H]+	C27H30O14	482-38-2	Kaempferol 3,7-dirhamnoside(Kaempferitrin)	Flavonols
pme2954	3.03E+02	1.19E+02	3.02E+02	[M+H]+	C15H10O7	117-39-5	Quercetin	Flavonols
pme3211	4.63E+02	6.20E+01	4.64E+02	[M-H]-	C21H20O12	21637-25-2	Quercetin 3-O-glucoside(Isotrifoliin)	Flavonols
pmn001583	6.09E+02	2.90E+01	6.10E+02	[M-H]-	C27H30O16	52525-35-6	Bioquercetin	Flavonols
pmn001614	5.77E+02	1.56E+02	5.78E+02	[M-H]-	C27H30O14	-	Kaempferol-3,7-O-α-L-rhamnoside	Flavonols
pmn001637	4.17E+02	1.17E+02	4.18E+02	[M-H]-	C20H18O10	5041-67-8	Juglanin	Flavonols
pmn001641	4.75E+02	1.58E+02	4.76E+02	[M-H]-	C22H20O12	-	Kaempferide 3-O-β-D-glucuronide	Flavonols
pmn001642	5.03E+02	1.54E+02	5.04E+02	[M-H]-	C23H20O13	-	Kaempferol 3-O-β-(2''-O-acetyl-β-D-glucuronide)	Flavonols
pmn001644	5.19E+02	1.22E+02	5.20E+02	[M-H]-	C23H20O14	-	Quercetin 3-O-β-(2''-O-acetylβ-D-glucuronide)	Flavonols
pmn001645	5.33E+02	6.40E+01	5.34E+02	[M-H]-	C24H22O14	-	Isorhamnetin 3-O-β-(2''-O-acetyl-β-D-glucuronide)	Flavonols
pmp000589	5.51E+02	3.30E+01	5.50E+02	[M+H]+	C24H22O15	-	Quercetin-7-O-(6'-O-malonyl)-β-D-glucoside	Flavonols
pmp000596	6.27E+02	1.20E+02	6.26E+02	[M+H]+	C27H30O17	-	Quercetin 3,7-bis-O-β-D-glucoside	Flavonols
pmp001309	4.65E+02	1.70E+01	4.64E+02	[M+H]+	C21H20O12	-	6-Hydroxykaempferol-7-O-glucoside	Flavonols
pmp001310	6.27E+02	1.60E+01	6.26E+02	[M+H]+	C27H30O17	-	6-Hydroxykaempferol-3,6-O-Diglucoside	Flavonols
pmp001311	6.27E+02	9.80E+01	6.26E+02	[M+H]+	C27H30O17	-	6-Hydroxykaempferol-7,6-O-Diglucoside	Flavonols
pmp001312	7.89E+02	9.60E+01	7.88E+02	[M+H]+	C33H40O22	-	6-Hydroxykaempferol-3,7,6-O-triglycoside	Flavonols
pmp001314	7.73E+02	9.70E+01	7.72E+02	[M+H]+	C33H40O21	-	6-Hydroxykaempferol-3-O-rutin-6-O-glucoside	Flavonols

mws0063	2.71E+02	1.47E+02	2.70E+02	[M+H]+	C15H10O5	446-72-0	Genistein	Isoflavones
mws0918	2.83E+02	3.90E+01	2.84E+02	[M-H]-	C16H12O5	552-59-0	Prunetin	Isoflavones
mws2118	4.35E+02	1.16E+02	4.36E+02	[M-H]-	C21H24O10	60-81-1	Phloretin 2'-O-glucoside	Isoflavones
pme3285	2.75E+02	1.80E+01	2.74E+02	[M+H]+	C15H14O5	2545-00-8	Afzelechin(3,5,7,4'-Tetrahydroxyflavan)	Isoflavones
pmp000194	5.19E+02	1.11E+02	5.18E+02	[M+H]+	C24H22O13	-	Malonyglygenistin	Isoflavones
pmp000409	4.31E+02	1.30E+02	4.30E+02	[M+H]+	C22H22O9	486-62-4	Ononin	Isoflavones
mws0836	5.77E+02	7.00E+01	5.78E+02	[M-H]-	C30H26O12	20315-25-7	Procyanidin B1	Proanthocyanidins
pmb0837	5.77E+02	1.83E+02	5.76E+02	[M+H]+	C30H24O12	86631-39-2	Procyanidin A3	Proanthocyanidins
pmb2586	5.93E+02	1.31E+02	5.94E+02	[M-H]-	C30H26O13	-	Gallocatechin-catechin	Proanthocyanidins
pmb2947	8.65E+02	1.51E+02	8.66E+02	[M-H]-	C45H38O18	-	Catechin-catechin-catechin	Proanthocyanidins
pmb3114	5.61E+02	1.06E+02	5.62E+02	[M-H]-	C30H26O11	-	Epicatechin-epiafzelechin	Proanthocyanidins
pme0431	5.77E+02	1.82E+02	5.76E+02	[M+H]+	C30H24O12	12798-56-0	Procyanidin A1	Proanthocyanidins
pme0434	5.77E+02	7.10E+01	5.78E+02	[M-H]-	C30H26O12	29106-49-8	Procyanidin B2	Proanthocyanidins
pmn001646	8.65E+02	1.84E+02	8.66E+02	[M-H]-	C45H38O18	37064-30-5	Procyanidin C1	Proanthocyanidins
pmn001649	1.15E+03	1.49E+02	1.15E+03	[M-H]-	C60H50O24	86631-38-1	Cinnamtannin A2	Proanthocyanidins
pmn001650	1.15E+03	1.50E+02	1.15E+03	[M-H]-	C60H50O24	88038-12-4	Cinnamtannin B2	Proanthocyanidins
pmn001667	5.77E+02	7.30E+01	5.78E+02	[M-H]-	C30H26O12	29106-51-2	Procyanidin B4	Proanthocyanidins
pmp000100	8.67E+02	1.09E+02	8.66E+02	[M+H]+	C45H38O18	79763-28-3	Arecatannin B1	Proanthocyanidins
pmp000101	8.67E+02	1.10E+02	8.66E+02	[M+H]+	C45H38O18	87727-69-3	Arecatannin C1	Proanthocyanidins
pmp000102	1.16E+03	1.07E+02	1.15E+03	[M+H]+	C60H50O24	-	Arecatannin A1	Proanthocyanidins
pmp000103	1.16E+03	1.08E+02	1.15E+03	[M+H]+	C60H50O24	79763-29-4	Arecatannin A2	Proanthocyanidins
pmp000274	5.79E+02	7.20E+01	5.78E+02	[M+H]+	C30H26O12	23567-23-9	Procyanidin B3	Proanthocyanidins
pmp000280	8.67E+02	1.85E+02	8.66E+02	[M+H]+	C45H38O18	37064-31-6	Procyanidin C2	Proanthocyanidins

Note: [M]+ means that the substance itself is easy to form charged ions; [M+H]+ means the substance added a proton during detection; [M-H]- means the substance lost a proton during detection; [M-Cl]+ means that the purchased compound was chloride, but there was no ion peak of chloride ion during detection.
